# Molecular detection of a novel *Ancylostoma* sp. by whole mtDNA sequence from pangolin *Manis javanica*

**DOI:** 10.1186/s13071-022-05191-0

**Published:** 2022-03-02

**Authors:** Merga Daba Tuli, Hongyi Li, Song Li, Junqiong Zhai, Yajiang Wu, Wanyi Huang, Yaoyu Feng, Wu Chen, Dongjuan Yuan

**Affiliations:** 1 Center for Emerging and Zoonotic Diseases, College of Veterinary Medicine, South China Agricultural University, Guangzhou, 510642 China; 2 Guangdong Laboratory for Lingnan Modern Agriculture, Guangzhou, 510642 China; 3 Guangzhou Zoo & Guangzhou Wildlife Research Center, Guangzhou, 510070 China

**Keywords:** *Ancylostoma* sp., Ancylostomatidae, Mitochondrial genome, Phylogenetic analysis

## Abstract

**Background:**

*Ancylostoma* species are hematophagous parasites that cause chronic hemorrhage in various animals and humans. Pangolins, also known as scaly anteaters, are mammals that live in soil environments where they are readily exposed to soil-borne parasitic nematodes. However, only a limited number of helminth species have been identified in this animal host so far.

**Methods:**

*Ancylostoma* sp. was isolated from a wild pangolin, and the complete mitochondrial (mt) genome of *Ancylostoma* sp. was obtained by Illumina sequencing of total genomic DNA.

**Results:**

The circular complete mt genome that was assembled had a total length of 13,757 bp and comprised 12 protein-coding genes (PCGs), 22 transfer ribosomal RNAs, two ribosomal RNAs (rRNAs), two non-coding regions and one AT-rich region, but lacked the gene coding for ATPase subunit 8 (*atp8*). The overall AT content of the mt genome of *Ancylostoma* sp. was 76%, which is similar to that of other nematodes. The PCGs used two start codons (ATT and TTG) and three stop codons (TAA, TAG, and T). The nucleotide identity of the 12 PCGs ranged from 83.1% to 89.7% and had the highest sequence identity with *Ancylostoma caninum* among species in the Ancylostomatidae family. Also, the pangolin-derived *Ancylostoma* sp. lacked repeat sequences in the non-coding regions and in the unique sequence of the short non-coding regions, which differentiated it from other *Ancylostoma* species. In addition, phylogenetic analyses of 18S rRNA and mtDNA sequences revealed that the *Ancylostoma* sp. was positioned in a separate branch in the subfamily Ancylostomatinae along with other *Ancylostoma* species.

**Conclusions:**

The *Ancylostoma* sp. isolated from a pangolin in this study was identified as a possible new *Ancylostoma* species*.* The identification of this *Ancylostoma* sp. from pangolin enriches our knowledge of the species in the Ancylostomatidae family and provides information that will lead to a better understanding of the taxonomy, diagnostics, and biology of hookworms.

**Graphical Abstract:**

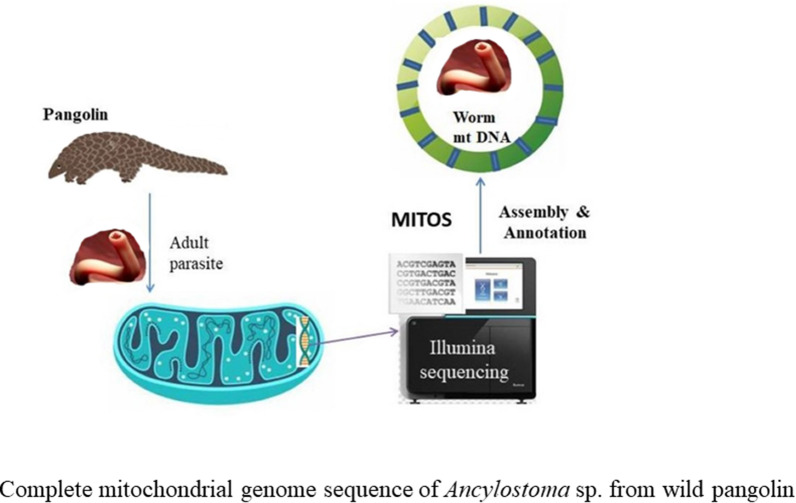

**Supplementary Information:**

The online version contains supplementary material available at 10.1186/s13071-022-05191-0.

## Background

Hookworms are hematophagous nematodes of mammals, and adult parasites reside in their host’s gastrointestinal tract, causing anemia, stunted growth, tissue damage, inflammation in dogs and cats, and significant neonatal mortality through transmission to unborn pups [1, 2]. *Ancylostoma* species are members of the family Ancylostomatidae and infect animals and humans by penetrating the skin or being ingested as third-stage larvae, via paratenic hosts, or by transplacental passage [3]. Although some *Ancylostoma* species have been identified in wild bears, hyenas, red foxes, raccoons and pandas by morphological investigation [4–10], newly emerging *Ancylostoma* species have not been well identified in other wild animals.

Morphological and morphometric methods have been used to classify nematodes based on the shape of their mouth, tail and sexual organ, the size of the worm body, eggs and larvae [11, 12]. However, these traditional methods for nematode identification have been challenged for a number of reasons. Firstly, some species share similar morphological characteristics; for example, eggs of *Necator americanus*, *Ancylostoma* species and Strongylids have similar shapes, and it is not easy to discriminate between closely related species [13]. Traditional identification methods also face some challenges in identifying cryptic species of parasitic nematodes due to their identical morphological features [6]. In addition, nematode collection is also complicated by seasonal fluctuations in the prevalence and intensity of specific species; consequently long-term monitoring is required to collect all nematodes of particular hosts [14]. Another complication is obtaining intact nematodes for morphological identification. Therefore, molecular approaches have been used to discriminate nematodes via nuclear genetic markers and mitochondrial genomes. The mitochondrial (mt) genome has important unique features of maternal inheritance and rapid evolution, but an absence of recombination [15, 16]. Hence, mt genomes provide genetic markers for molecular identification, epidemiological and genetic studies, as well as for phylogenetic and population studies [17–20].

Pangolins, also known as the scaly anteater, are endangered and rare animals that require special protection [21]. These small mammals live in soil environments and can be easily exposed to soil parasitic nematodes. However, only a few helminth parasites have been identified, using egg and adult morphological characteristics, after being isolated from the pangolin gastrointestinal tract; many others are still unknown. A total of 13 parasitic helminths have been reported from pangolins to date. Of these, eight helminth parasites were isolated from the gastrointestinal tract in egg, larvae and adult morphological investigations, including *Cylicospirura* sp., *Leipernema leiperi*, *Manistrongylus meyeri*, *Necator americanus*, *Strongyloides* sp., *Trichochenia meyeri*, *Ancylostoma* sp. and *Gendrespirura* sp. [22–27]*.* Until recently, the identification of *Ancylostoma* species in pangolin was limited to the genus level. In the family Ancylostomatidae, only *N. americanus* has been identified in pangolin to the species level [28]. However, there is a paucity of molecular data for identifying *Ancylostoma* species in pangolins. The aims of this study were to obtain a molecular characterization of a novel *Ancylostoma* sp. originated from a wild pangolin through the sequencing of total DNA using the Illumina sequencing platform (Illumina, Inc., San Diego, CA, USA).

## Methods

### Parasite collection

Guangzhou customs confiscated two pangolins from poachers and placed them in the Guangzhou Zoo, Guangdong Province, China. No information on the origin and species of the pangolins was available. One pangolin suffered severe trauma and a purulent infection of the forelimb and ultimately died due to complicated infections. During the post-mortem examination, a total of 15 adult parasites were collected from the duodenum of the naturally infected pangolin. The parasites were washed completely in phosphate- buffered saline, preserved in 70% ethanol and frozen for further identification. Prior to examination under a microscrope, the worms were cleaned with lactophenol and mounted in glycerine. We examined several frozen worms to obtain a complete description of their morphological features under dissecting microscopes (magnifications: 10–40×) and light microscopes (magnifications: 40–100×), but it was difficult to obtain precise morphological features.

### DNA extraction and whole-genome amplification

Total genomic DNA was extracted from a single adult worm using the Wizard® SV Genomic DNA Purification System (Promega, Guangzhou, China) according to the manufacturer's instructions and then stored at − 20 °C until use. Complete genomic DNA was amplified using a whole genome amplification kit (REPLI-g® Midi Kit; Qiagen, Hilden, Germany). All procedures were performed according to the manufacturer’s instructions. The amplified DNA was sequenced with an Illumina Novaseq 6000 sequencing platform using a 150-bp paired-end technique (Illumina, Inc.). Approximately 12 Gb of sequence data had a quality score (*Q*-score) ≥ 20.

### PCR amplification and DNA sequencing

The 18S ribosomal ribonucleic acid (rRNA) gene was amplified from the total extracted DNA of the observed worm using DreamTaq DNA Polymerase with the primers NC18S (AAAGATTAAGCCATGCA) and NC5B (GCAGGTTCACCTACAGAT) [29]. The amplification procedure was: 95 °C for 5 min; followed by 35 cycles of 95 °C for 30 s, 54 °C for 30 s, 71 °C for 75 min and 72 °C for 5 min. The amplified fragments were visualized and verified by electrophoresis in a 1.5% agar gel (Sangon Biotech Co., Ltd. Shanghai, China) with staining (0.2 mg/ml ethidium bromide). The PCR fragments were sequenced by Sanger sequencing (Sangon Biotech Co.).

### Assembly of the complete mt genome of pangolin and worm

The raw data was mapped to the pangolin genome and then filtered using Samtools (v1. 7) to remove the host gene sequences [30]. The filtered data were assembled into contigs and scaffolds using SPAdes (v3.14.1) [31]. Contigs were aligned into the nucleotide (nt) database using BLAST+ (v2.11.0) [32]. We extracted contigs that contained worm mt genomes with a sequencing depth > 100 and a length > 150 bp. Finally, eight contigs were randomly chosen as a seed sequence, and each seed sequence was assembled using Novoplasty (v.4.2) to reconstruct the complete mt genome of the worm [33]. To determine host identity, the filtered host data were also assembled into contigs and scaffolds using SPAdes, and all the mitochondrial contigs were aligned to the nt database using BLAST+ (v2.11.0). We identified the pangolin mtDNA by comparing it with the known mtDNA of pangolin species available in GenBank.

### Gene annotation and sequence analysis

Gene annotation of the assembled mt genome was conducted using MITOS and Geseq (https://chlorobox.mpimp-golm.mpg.de/geseq.html) [34]. The Mitos webserver was employed to predict protein-coding genes (PCGs) and non-coding regions (NCRs) of parasitic nematodes using the genetic code of invertebrate mtDNA (http://mitos.bioinf.uni-leipzig.de) [35]. Initiation and termination codons were identified using the Expasy translation tool (https://web.expasy.org/translate/) [36]. The secondary structures of transfer RNA (tRNA) were predicted and shown by MiTFi and the webserver FoRNA on Mitos [37]. Both rRNA genes (small and large ribosomal subunits [*rrnS* and *rrnL*, respectively]) were identified by MiTFi. The codon usage of amino acids for PCGs was determined by the sequence manipulation suite [38]. The complete mt genome was visualized by the MTviz (http://pacosy.informatik.uni-leipzig.de/mtviz/). A comparison of the nucleotide identity (%) of the observed worm mt genome with 13 closely related species of the Ancylostomatidae family was conducted using Clustal Omega [39].

### Phylogenetic analysis of 18S rRNA and PCGs of mt genome of worm

We obtained 18S rRNA sequences of 14 nematodes from the NCBI database and used these and the amplified 18S rRNA of the worm to construct a phylogenetic tree (Additional file 1: Table S1). The maximum likelihood (ML) method was performed to evaluate the phylogenetic tree, and the ML tree was made with the TPM3 + G4 model using RAxML-ng (v. 1.0.2) [40]. ML bootstrap > 70% was considered to be strong support [41].

We obtained nucleotide sequences of 12 PCGs from the mt genome of the worm isolated from the pangolin. We also downloaded the complete mt genome sequences of 13 species in the Ancylostomatidae family and 4 species in the Chabertiidae family (outgroup) from NCBI GenBank and aligned these for sequence comparison (Additional file 1: Table S2). A phylogenic tree was reconstructed with RAxML-ng (v. 1.0.2) and a ML method was used with the GTR+G+I model.

## Results

### Identification of pangolin species

To identify the pangolin species implicated in this case, we obtained the mt genome of the animal, with a total length of 16,574 bp, from Illumina sequencing data. This mtDNA showed the highest sequence identity (99.50%) and coverage (99.0%) with *Manis javanica* (Malayan pangolin) available from GenBank (accession number: MG196302.1).

### Observation on the worm

The worms were isolated from the wild pangolin's duodenum and frozen immediately in 75% ethanol for further identification. The worms were round and tapered at both ends. However, it was challenging to observe precise morphological features due to frozen state of the worms. Therefore, we performed molecular characterization using total genomic DNA by Illumina sequencing.

### Primary identification of worm by molecular markers

**Fig. 1:**
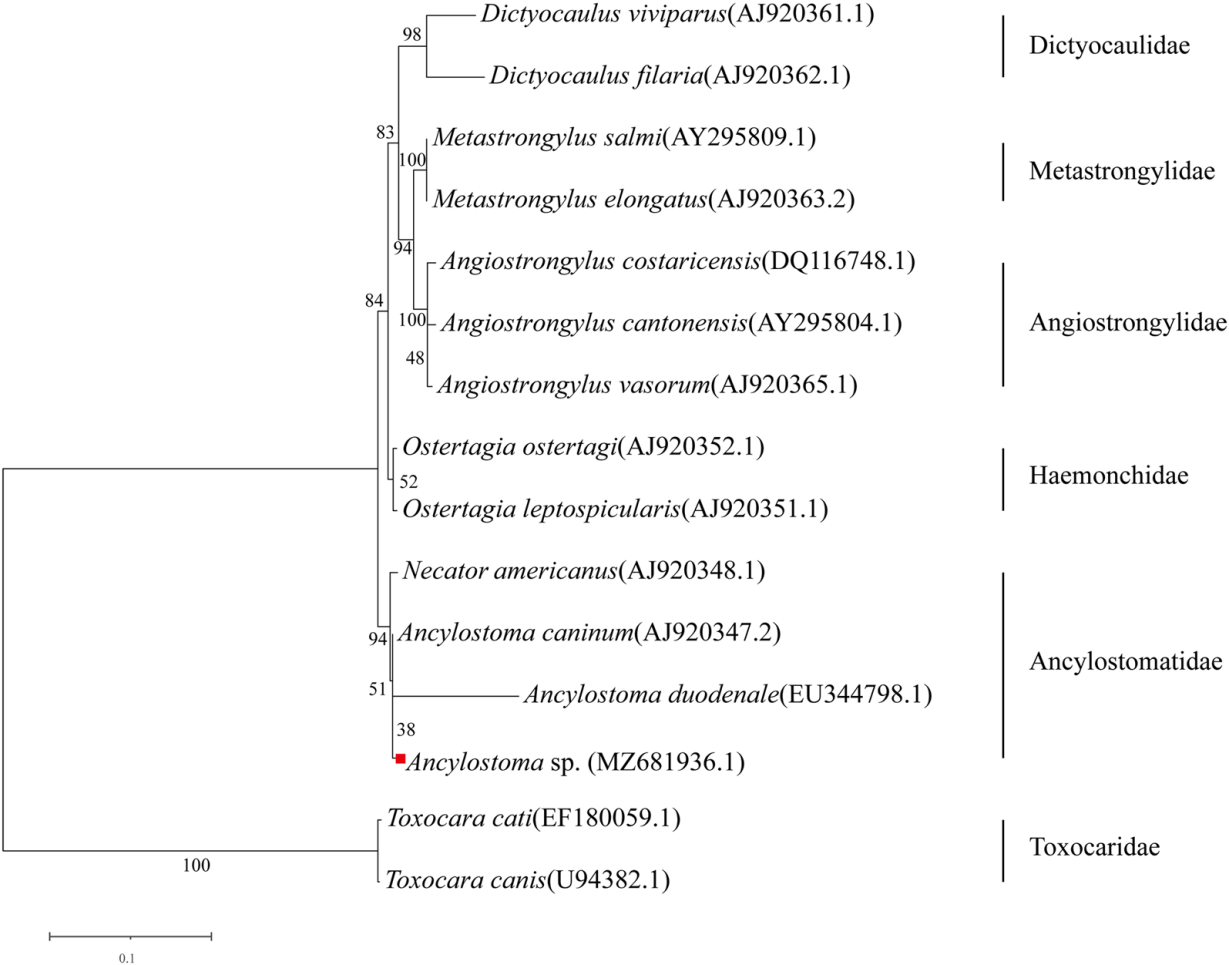
Phylogenetic tree of 18S rDNA sequences from *Ancylostoma* sp. and species of the orders Strongylida and Ascaridida. The phylogenetic relationship of this tree is inferred using the maximum likelihood (ML) method and order Ascaridida as outgroup (*Toxocara cati* and *Toxocara canis*). Bootstrap values are shown in the nodes. Scale bar represents the number of nucleotide substitutions per site

The amplified 18S rDNA sequence of the worm was 1681 bp and was deposited in GenBank databases under accession number: MZ681936.1. It showed 99.88% sequence identity with the 18S rDNA sequence of *A. caninum* from GenBank (accession number: AJ920347.2). Phylogenetic analysis of 18S rRNA sequences showed that the amplified 18S rDNA sequence of the worm clustered with *Ancylostoma duodenale*, *A. caninum* and *N. americanus* in the family of Ancylostomatidae (Fig. 1). This worm was relatively closer to *Ancylostoma* species than *N. americanus*. Thus, we proposed that this worm might be closely related to *Ancylostoma* species in the Ancylostomatidae family.

### Features, gene organization and composition of the mt genome

**Fig. 2:**
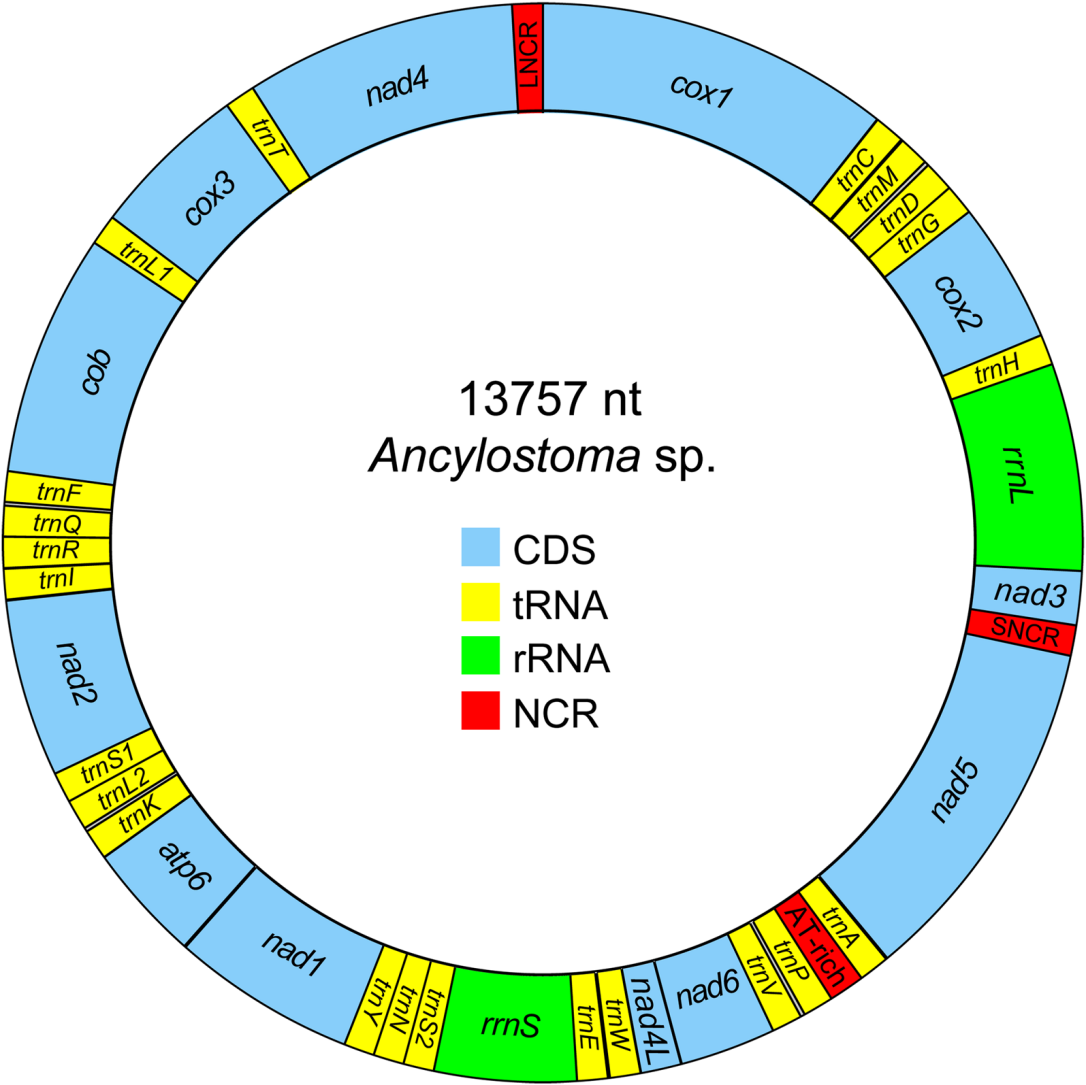
Mitochondrial genome organization of *Ancylostoma* sp. The map shows 12 PCGs, 22 tRNAs (shown as abbreviations with the initial letter of their amino acids) and 2 rRNAs. Each of the 2 leucines (L1 and L2) is identified for the codon families CUN and UUR, respectively, and each of the 2 serines (S1 and S2) is identified for the codon families AGR and UGN, respectively. Inner circle indicates GC content of the mt genome.* Abbreviations*: CDS, coding DNA sequence; LNCR, long non-coding region; SNCR, short non-coding region

For further identification of this worm, we obtained 12 Gb of raw data with 80,271,718 reads from the complete genomic DNA of the worm using Illumina sequencing. The assembled sequence showed that the complete mt genome of the worm was 13,757 bp; this sequence was deposited in GenBank with accession number MZ665481.1. The mt genome of this worm was a circular DNA molecule and contained 36 genes, comprising 12 PCGs, 22 tRNA genes (2 coding for leucine and 2 coding for serine), two rRNA genes, two NCRs (a long non-coding region [LNCR] and a short non-coding region [SNCR]) and an AT-rich region. Interestingly, the ATPase subunit 8 gene (*atp8*) was missing from the mt genome (Fig. 2). Twelve PCGs of this worm were transcribed in the same direction. In general, the overall base composition of the mt genome of this worm was: A = 27%, T = 49%, C = 7% and G = 17%, with an entire AT content of 76%, which was greatly inclined towards A and T bases. The AT- and GC-skews of the worm’s mt genome were determined to be: AT-skew (A−T)/(A+T) = − 0.26; GC-skew (G−C)/(G+C) = 0.41; Additional file 1: Table S3).

### PCGs and codon usage

The total length of the 12 PCGs was 10,283 bp, which accounts for 74.7% of the entire mt genome of the worm. These PCGs ranged in size from 234 bp of NADH dehydrogenase subunit 4L (*nad4L*) to 1578 bp of cytochrome* c* oxidase subunit I (*cox1*). The overall base composition of the PCGs in the worm mt genome was: A = 25%, T = 50%, C = 7% and G = 18%, with AT skew = − 0.32 and GC skew = 0.42, which was largely biased towards the A and T bases. The most favored nucleotide was the T base, but the C base was the least favored in PCGs of the worm. The *nad4L* gene had the highest AT content (81%) among the 12 PCGs, while *cox1* had the lowest AT content (68%) (Additional file 1: Table S3). All of the AT-skew values of the 12 PCGs were negative, and all of the GC-skew values were positive.

The PCGs of the worm contained a total of 3417 amino acids. Two different types of codons (ATT and TTG) were used as start codons, while three different codons (TAA, TAG and T) were used as stop codons (Table 1). ATT was used as a start codon in 10 genes, namely *cox1*, *cox2*, *nad3*, *nad5*, *nad6*, *nad4L*, *nad1*, *atp6*, *cob* and *cox3*, while TTG was used as a start codon in the *nad2* and *nad4* genes. TAA was used as a stop codon in seven genes: *cox1*, *cox2*, *nad6*, *nad4L*, *nad1*, cytochrome b (*cob*) and *nad4*. TAG was used as a stop codon in three genes, including *nad3*, *atp6* and *nad2*; moreover, an incomplete codon (T) was used in the genes *nad5* and *cox3* for transcription termination. Thus, in 12 PCGs, ATT and TAA were the most frequently used start and stop codons, respectively. Phenylalanine (TTT: 13.0%) was the most repeatedly employed amino acid in the mt genome of the worm, followed by leucine (TTA: 8.6%) and isoleucine (ATT: 7.0%). However, some transcription codons were absent, such as CGC and CGG coding for arginine and CTC coding for leucine (Table 2).

**Table 1:** Mitochrondrial genome organization, showing start and stop codons of PCGs and as anticodons of tRNA of *Ancylostoma* sp.

Gene/region	Position	Length		Codon		Anticodon
	Start to end	Number of nucleotides	Number of amino acids	Start	Stop	
*cox1*	1–1578	1578	525	ATT	TAA	
*trnC*	1577–1630	54				GCA
*trnM*	1637–1695	59				CAT
*trnD*	1714–1772	59				GTC
*trnG*	1773–1829	57				TCC
*cox2*	1830–2525	696	231	ATT	TAA	
*trnH*	2526–2581	56				GTG
*rrnL*	2582–3548	967				
*nad3*	3549–3884	336	111	ATT	TAG	
SNCR	3885–3984	100				
*nad5*	3985–5560	1576	525	ATT	T	
*trnA*	5567–5622	56				TGC
AT-rich	5623–5883	261				
*trnP*	5884–5938	55				TGG
*trnV*	5957–6011	55				TAC
*nad6*	6012–6446	435	144	ATT	TAA	
*nad4L*	6450–6683	234	77	ATT	TAA	
*trnW*	6686–6742	57				TCA
*trnE*	6751–6809	59				TTC
*rrnS*	6810–7507	698				
*trnS2*(UGN)	7508–7561	54				TGA
*trnN*	7562–7617	56				GTT
*trnY*	7618–7673	56				GTA
*nad1*	7674–8546	873	290	ATT	TAA	
*atp6*	8553–9152	600	199	ATT	TAG	
*trnK*	9152–9214	63				TTT
*trnL2*(UUR)	9231–9285	55				TAA
*trnS1*(AGR)	9286–9338	53				TCT
*nad2*	9339–10,184	846	281	TTG	TAG	
*trnI*	10,189–10,247	59				GAT
*trnR*	10,251–10,304	54				ACG
*trnQ*	10,306–10,360	55				TTG
*trnF*	10,376–10,432	57				GAA
*cob*	10,433–11,545	1113	370	ATT	TAA	
*trnL1*(CUN)	11,546–11,600	55				TAG
*cox3*	11,601–12,366	766	255	ATT	T	
*trnT*	12,367–12,421	55				TGT
*nad4*	12,422–13,651	1230	409	TTG	TAA	
LNCR	13,652–13,757	106				

LNCR, Long non-coding region; SNCR, short non-coding region; see Abbreviations for other abbreviations and gene names

**Table 2:** Amino acid codons and percentage of codon usage for PCGs in the *Ancylostoma* sp. mt genome

Amino acid	Codon	Number	Frequency (%)	Amino acid	Codon	Number	Frequency (%)
Ala	GCA	24	0.70	Leu	TTA	297	8.67
Ala	GCC	7	0.20	Leu	TTG	209	6.10
Ala	GCG	6	0.18	Lys	AAA	50	1.46
Ala	GCT	61	1.78	Lys	AAG	57	1.66
Arg	CGA	2	0.06	Met	ATA	124	3.62
Arg	CGC	0	0.00	Met	ATG	110	3.21
Arg	CGG	0	0.00	Phe	TTC	18	0.53
Arg	CGT	29	0.85	Phe	TTT	446	13.01
Asn	AAC	9	0.26	Pro	CCA	7	0.20
Asn	AAT	132	3.85	Pro	CCC	5	0.15
Asp	GAC	9	0.26	Pro	CCG	11	0.32
Asp	GAT	56	1.63	Pro	CCT	57	1.66
Cys	TGC	2	0.06	Ser	AGA	57	1.66
Cys	TGT	40	1.17	Ser	AGC	7	0.20
End	TAA	7	0.20	Ser	AGG	43	1.25
End	TAG	3	0.09	Ser	AGT	119	3.47
Gln	CAA	21	0.61	Ser	TCA	47	1.37
Gln	CAG	21	0.61	Ser	TCC	4	0.12
Glu	GAA	35	1.02	Ser	TCG	9	0.26
Glu	GAG	42	1.23	Ser	TCT	92	2.68
Gly	GGA	12	0.35	Thr	ACA	30	0.88
Gly	GGC	6	0.18	Thr	ACC	1	0.03
Gly	GGG	33	0.96	Thr	ACG	17	0.50
Gly	GGT	139	4.06	Thr	ACT	62	1.81
His	CAC	12	0.35	Trp	TGA	48	1.40
His	CAT	42	1.23	Trp	TGG	20	0.58
Ile	ATC	10	0.29	Tyr	TAC	11	0.32
Ile	ATT	240	7.00	Tyr	TAT	171	4.99
Leu	CTA	6	0.18	Val	GTA	68	1.98
Leu	CTC	0	0.00	Val	GTC	10	0.29
Leu	CTG	9	0.26	Val	GTG	55	1.60
Leu	CTT	8	0.23	Val	GTT	142	4.14

### rRNA and tRNA genes

The worm had two rRNAs, including a large subunit (*rrnL*) of 967 bp and a small subunit (*rrnS*) of 698 bp*.* The *rrnL* was situated between *trnH* and *nad3*, while *rrnS* was found between *trnE* and *trnS2*. The position of rRNA in *Ancylostoma* sp. was similar to that found in other *Ancylostoma* species but distinct from that found in *Trichinella spiralis *(class Adenophorea) [43]. The *rrnL* of the worm was longer than the *rrnL* of 13 species in the Ancylostomatidae family, which ranged from 957 bp (*Uncinaria sanguinis*) to 963 bp (*A. caninum*) (Table 3). In addition, sequence identity of *rrnL* and *rrnS* in the observed worm was higher with species in the subfamily Ancylostomatinae than with species in the subfamily Bunostominae. The highest sequence identity of *rrnL* of the worm was 89.6% with *Ancylostoma tubaeforme* compared to other species in the Ancylostomatidae family, and *rrnS* had the highest sequence identity of 94% with *Ancylostoma ceylanicum* (Table 3).

**Table 3:** Comparisons of nucleotide identity of PCGs, rRNA and NCRs of the mt genome of *Ancylostoma* sp. with the mt genomes of other Ancylostomatidae species

Region	Gene/region	Length of gene regions (bp)/nucleotide identity (%)													
		*Ancylostoma* sp. (MZ665481.1)	Ancylostomatinae						Bunostominae						
			*Ancylostoma caninum*		*Ancylostoma ceylanicum*		*Ancylostoma duodenale*	*Ancylostoma tubaeforme*	*Uncinaria sanguinis*		*Nector americanus*	*Bunostomum phlebotomum*		*Bunostomum trigonocephalum*	
			AP017673.1	FJ483518.1	KY640299.1	AP017674.1	AJ417718.1	KY070315.1	KF924756.1	KF924757.1	AJ417719.2	MW067147.1	FJ483517.1	JQ234674.1	KF255998.1
		Length (bp)													
PCGs	*cox1*	1578	1578/87.6	1578/87.5	1578/87.9	1578/87.7	1577/87.9	1578/87.9	1578/86.1	1578/86.7	1575/86.6	1573/86.6	1573/87.0	1573/84.0	1576/84.3
	*cox1*	696	696/88.6	696/88.7	696/85.8	696/86.0	696/87.7	696/88.2	696/84.3	696/84.9	696/83.9	696/83.9	696/83.9	696/43.0	696/77.0
	*nad3*	336	336/85.1	336/85.1	336/84.7	336/84.5	336/86.0	336/88.3	336/82.7	336/80.0	336/83.0	336/80.9	336/81.2	336/51.6	336/63.5
	*nad5*	1576	1582/85.3	1576/85.4	1579/85.5	1582/85.8	1579/84.6	1576/84.9	1582/81.0	1582/81.6	1582/81.8	1582/80.0	1582/79.7	1582/78.9	1582/78.9
	*nad6*	435	432/83.1	435/83.6	435/83.3	435/81.8	435/80.0	435/82.9	435/78.1	435/76.5	435/79.0	435/76.7	435/76.7	435/60.9	435/41.9
	*nad4L*	234	234/89.7	234/91.0	234/92.8	234/91.8	234/91.8	234/90.6	234/86.3	234/86.3	234/88.8	234/88.4	234/88.4	234/78.1	234/47.3
	*nad1*	873	873/86.9	873/85.4	873/85.5	873/85.4	873/86.0	873/86.8	873/83.6	873/84.4	873/82.3	876/80.3	876/78.5	873/44.0	873/78.5
	*atp6*	600	600/89.1	600/88.8	599/89.8	602/89.6	599/90.1	600/88.6	600/82.0	600/83.6	598/84.6	600/83.8	600/84.1	600/57.6	600/75.6
	*nad2*	846	846/83.1	846/83.1	846/83.8	846/83.4	846/83.1	846/82.2	846/78.4	846/77.9	846/79.5	849/76.7	849/76.1	849/70.1	849/62.9
	*cob*	1113	1113/88.1	1113/88.1	1113/87.0	1113/86.8	1112/88.2	1113/87.9	1113/82.2	1113/81.6	1113/83.1	1113/83.9	1113/83.5	1113/78.2	1113/75.8
	*cox3*	766	766/89.3	766/89.3	766/87.7	766/87.9	766/88.5	766/87.7	766/86.0	766/84.4	766/86.6	766/85.9	766/86.5	766/81.0	766/81.2
	*nad4*	1230	1230/86.9	1230/86.2	1230/86.5	1230/86.7	1230/85.7	1230/87.4	1230/83.0	1230/81.8	1230/82.6	1230/80.4	1230/80.6	1230/78	1230/72.0
rRNA	*rrnL*	967	958/88.3	963/87.8	960/89.0	960/88.4	958/87.3	958/89.6	957/86.2	958/85.4	958/80.1	961/82.7	961/82.6	961/76.8	961/69.7
	*rrnS*	698	695/92.9	694/93.0	694/94.0	694/93.9	697/91.8	697/93.1	699/87.7	697/88.2	699/86.6	694/86.1	694/86.1	693/84.5	696/64.5
NCR	SNCR	100	88/null	87/42.1	61/null	66/null	79/null	88/57.1	81/41.6	82/null	67/null	24/65.0	21/null	2/null	2/null
	LNCR	106	100/70.7	100/68.6	103/70.5	94/52.3	109/78.2	107/73.0	86/73.2	86/73.2	73/null	108/58.3	106/43.9	100/48.1	106/42.8
AT-rich		261	262/76.4	272/80.8	244/80.3	246/78.3	268/74.5	286/76.8	333/73.2	331/74.9	173/null	235/50.6	234/52.3	219/56.0	218/60.0
CmtG		13,757	13,702	13,717	13,660	13,655	13,721	13,730	13,74	13,75	13,605	13,799	13,790	13,764	13,771
TI (%)			87.3	87.2	86.8	86.9	86.8	87.1	83.5	83.2	83.7	80.9	80.8	82.7	82.7

CmtG, Complete mitochondrial genome; TI, total identity, null, no identity

The length of the 22 tRNAs ranged from 53 bp (*trnS1*) to 63 bp (*trnS2* and *trnK*). The total length of the 22 tRNAs of the worm was 1239 bp with an A+T content of 80%; consequently, most codons were composed of A+T bases relative to G+C bases. Apart from serine (CUN and UUR) and leucine (AGR and UGN), there was a one-to-one binding between codon and anticodon for all other tRNAs. With the exception of *trnS1* and *trnS2*, all tRNA secondary structures of the mt genome of *Ancylostoma* sp. had the DHU arm and DHU loop, which were similar to those of most nematodes, including *Toxocara canis*, *Ascaris suum*, *A. tubaeforme*, *Onchocerca volvulus* and *Anisakis simplex* [44–48]. Only *trnI*, *trnK*, *trnS1* and *trnS2* had a pseudouridine (TΨC) arm. Other tRNAs lacked a pseudouridine (TΨC) arm and changed into a TV replacement loop. Moreover, an undeveloped form of the TΨC loop was only found in *trnK*; a typical TΨC loop was detected in *trn*M but it lacked TΨC arm (Additional file 1: Fig. S1).

### NCR and AT-rich regions

The LNCR of the worm was located between *nad4* and *cox1* with a length of 106 bp, whereas the SNCR was found between *nad3* and *nad5* with a length of 100. The entire base composition of the NCRs was as follows: A = 41%, G = 10%, C = 4%, T = 45%, AT = 86% and GC = 14%. The NCRs of this worm lacked repeat sequences, unlike other *Ancylostoma* species, including *A. caninum*, *A. ceylanicum*, *A. tubaeforme* and *A. duodenale*. LNCR sequence identity of the worm was 52.3–78.2% with related species in the subfamily Ancylostomatinae and 42.8–58.3% with species in the Bunostominae subfamily, but there was no sequence identity with *N. americanus*. The LNCR of the observed worm had the highest nucleotide identity of 78.2% with *A. duodenale* from GenBank (accession number: AJ417718.1) [49]. Nonetheless, the SNCR of the worm had low identity with a few species in the family Ancylostomatidae, while there was no sequence identity with many species in the family of Ancylostomatidae (Table 3). Thus, the SNCR was the unique region in the mt genome of the worm based on nucleotide identity (Table 3).

The AT-rich region was situated between *trnA* and *trnP* in the mt genome of the worm. The size of AT-rich region of the worm (261 bp) lay within range 173 bp (*N. americanus*) and 333 bp (*A. duodenale* and *B. phlebotomum*) (Table 3). The AT-rich region had 90% A+T content and comprised a poly-A stretch, poly-T stretch and microsatellites (such as an TA or TA repeat). The AT-rich region of the worm had a sequence identity of 73.2–80.8% with that of species in the subfamily Ancylostomatinae, and 50.6–60.0% sequence identify with some species in the subfamily Bunostominae. The AT-rich region of the worm had no sequence identity with that of *N. americanus* in the subfamily Bunostominae (Table 3). Thus, the sequence of the AT-rich region showed that this worm was more closely related to species in the subfamily Ancylostomatinae than to species in the subfamily Bunostominae.

### Comparison of the worm mt genome with that of species in the family Ancylostomatidae

Total sequences of the worm mt genome had higher identities of 86.8–87.3% with those of related species in the subfamily Ancylostomatinae than with those in the subfamily Bunostominae (Table 3). Moreover, the entire mt genome of the worm had the highest sequence identity of 87.3% with *A. caninum* compared to other Ancylostomatidae species (Table 3)*.* The relatively low sequence identity was noted with the *Bunostomum* species, *Uncinaria sanguinis,* and *N. americanus,* with sequence identity ranging from 80.8% to 83.7%. In PCGs, the most conserved gene across the subfamily Ancylostomatinae was *nad4L*, with a sequence identity of 89.7–92.8%, whereas *nad6* was the least conserved gene with 80.0–83.6% sequence identity (Table 3). The 12 PCGs of the collected worm also had the highest sequence identity (83.1–91.0%) with *A. caninum* compared with other species from the subfamilies Ancylostomatinae and Bunostominae. These results suggest that the reported worm is an undescribed *Ancylostoma* sp. and genetically related closer to *A. caninum* than to other *Ancylostoma* species.

### PCGs of the mt genome based on phylogenetic analysis

**Fig. 3:**
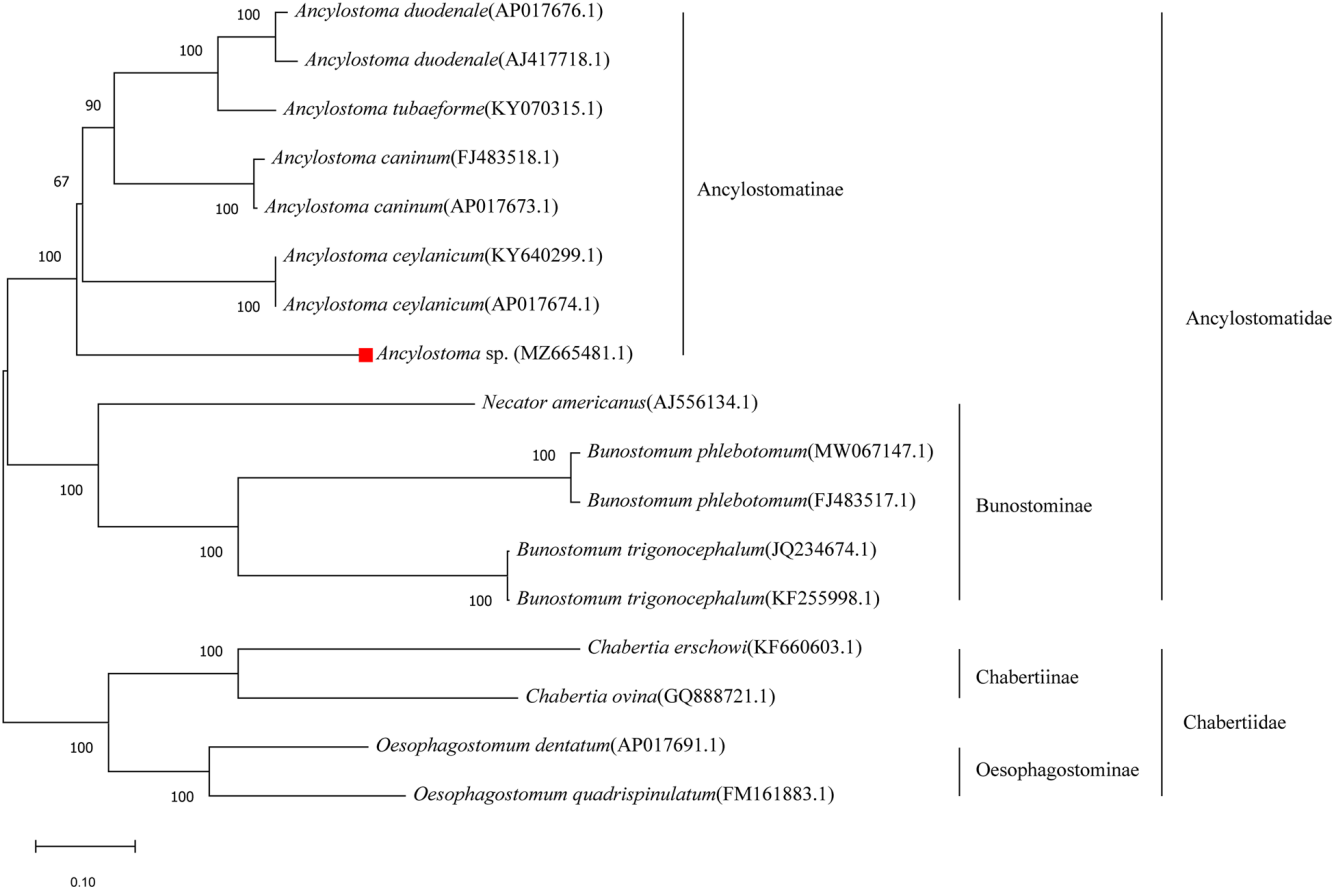
Phylogenetic tree of 12 PCG sequences from the mt genomes in the families of Ancylostomatidae, Chabertiidae and *Ancylostoma* sp. This tree is reconstructed based on the ML method. The numbers at the nodal points indicate the statistical values of the phylogenic tree. Nodal points indicate bootstrap values. Scale bar represents the number of nucleotide substitutions per site

The PCG sequences of the collected *Ancylostoma* sp., 12 species from the Ancylostomatidae family and 4 species from the Chabertiidae family (outgroup) were used to reconstruct the phylogenetic tree (Fig. 3). Accordingly, *Ancylostoma* sp. was grouped into the family Ancylostomatidae, separate from the species of the Chabertiidae family. In the Ancylostomatinae subfamily, *Ancylostoma* sp. was grouped with *A. ceylanicum*, *A. caninum*, *A. tubaeforme* and *A. duodenale*, while *N. americanus* and two *Bunostomum* species (*Bunostomum phlebotumum* and *Bunostomum trignocephalum*) were grouped in the Bunostominae subfamily (Fig. 3). Thus, the worm had a closer relationship with *A. ceylanicum, A. caninum*, *A. tubaeforme* and *A. duodenale* than to species in the subfamily Bunostominae. Phylogenetic analyses of the PCGs showed that *Ancylostoma* sp. clustered with other *Ancylostoma* species in the Ancylostomatinae subfamily. Sequence identity showed that the *Ancylostoma* sp. from the pangolin was distinct from known species of the genus *Ancylostoma*. Thus, the *Ancylostoma* analyzed herein may represent a novel species in the genus *Ancylostoma*.

## Discussion

*Ancylostoma* species are one of the most prevalent soil-transmitted helminths, affecting both domestic and wild animals, as well as humans. In this study, we identified a novel *Ancylostoma* sp. that originated from a Sunda pangolin (*Manis javanica*) by analysis of the mt genome using Illumina sequencing of total DNA.

The complete mt genome of *Ancylostoma* sp. was 13,757 bp, which is longer than that of *A*. *caninum* (13,717 bp) [50], *A. tubaeforme* (13,730 bp) [48], *A. ceylanicum* (13,660 bp) [51], *A*. *duodenale* (13,721 bp), *U. sanguinis* (13,753 bp) [52]*,* and *N. americanus* (13,606 bp), respectively [53], but shorter than that of *B. phlebotomum* (13,790 bp) [50]. This difference in mt genome length is due to the longer NCR and rRNA sequences of *A. caninum* in comparison to those of other Ancylostomatidae species. Thus, differences in mt genome size may be a useful indicator to increase our understanding of mtDNA mutation, mitochondrial genetics and evolutionary biology. The 12 PCGs of *Ancylostoma* sp. were transcribed in the same direction as those of class Secernentea nematodes of hookworms (*A. duodenale* and *N. americanus*) and other species (*Ascaris suum* and *Onchocerca volvulus*) [45, 49]. The direction of transcription in the mtDNA of Secernentea nematodes is conserved. The mt genome organization and gene arrangement of *Ancylostoma* sp. were similar with those of *N. americanus* and *A. duodenale*, with the exception of the position of *rrnL* and *rrnS*, which were located between *trnH* and *nad3*, and *trnE* and *trnS2*, respectively [49]. However, the gene arrangement and organization of *Ancylostoma* sp. were identical with those of *A. tubaeforme*, *A. caninum* and *B. phlebotomum* [48, 50]. The *cox1* gene in *Ancylostoma* sp. was the longest gene among the 12 PCGs, similar to the situation in *A. tubaeforme* [48]; conversely, *nad5* was the longest gene in *A. ceylanicum*, *A. doudenale* and *N. americanus* [48, 49, 53]. *Nad4L* was the shortest region of the PCGs in *Ancylostoma* sp., which is consistent with observations in other hookworms [48, 50]. The overall base composition of PCGs in *Ancylostoma* sp. was inclined towards AT bases. All PCGs from different nematodes have a higher AT base selection that maintains the stability of gene structure through decreasing gene mutation [54]. Thus, the length of mtDNA and PCGs of *Ancylostoma* sp. was slightly different from that of known *Ancylostoma* species. A complete mt genome sequence of *Ancylostoma* species can be used as a genetic marker for molecular investigation and diagnosis of members of the family Ancylostomatidae. Moreover, the entire mt genome data of *Ancylostoma* sp. would contribute to a further understanding of the pangolin helminth fauna.

ATT is the most common start codon found in hookworms, followed by TTG. Likewise, *Ancylostoma* sp. used ATT and TTG as start codons, similar to *A. ceylanicum* and *A. duodenale* [49, 51]. Nonetheless, *A. tubaeforme* and *A. caninum* utilize GTG as additional start codons [48, 50]. This variation in codon usage in the different genes of parasite species arises from various factors, but mainly from compositional constraints and translational selection [55]. It is noteworthy that the start codons of *nad5* and *nad6* in *Ancylostoma* species were remarkably different from other those of PCGs [8]. With the exception of *A. ceylanicum*, the *nad5* gene of this *Ancylostoma* sp. and other *Ancylostoma* species uses ATT as a start codon. Similarly, the *nad6* gene of *Ancylostoma* sp. utilized ATT as a start codon, consistent with *A. ceylanicum* but distinct from *A. tubaeforme* and *A. caninum,* both of which use GTG codons [48, 50]. *Ancylostoma* sp. utilized three codons (TAA, TAG and T) as stop codons, but *A. caninum*, *A. tubaeforme* and *A. doudenale* use additional TA codons [48–50]. The translation termination in the *cox3* and *nad5* genes of *Ancylostoma* sp. used an incomplete codon of T, which is similar to that of *cox3* and *nad5* genes in *A. ceylanicum* and *N. americanus* [51, 53]. It is believed that post-transcriptional polyadenylation has been shown to complete codons by adding A’s to incomplete stop codons, resulting in TAA [56].

The majority of codons were composed of A and T bases, contributing to the high AT content of the entire mt genome of *Ancylostoma* sp. Nucleotide bias significantly impacts codon usage and amino acid composition. For example, it has been reported that mutational bias at the nucleotide level can alter codon usage and amino acid content [57, 58]. The length of the *rrnL* gene of *Ancylostoma* sp. was 967 bp, which is longer than that of other known species of hookworm by 4 bp (*A. caninum*, *B. phlebotumum*), 7 bp (*A. ceylanicum*), 9 bp (*A. tubaeforme* and *N. americanus*) and 11 bp (*A. doudenale*) [48–50]. The *rrnS* gene of *Ancylostoma* sp. (698 bp) was slightly longer than that of other hookworm species, with the exception of *N. americanus* (699 bp) [49]. Thus, the difference in the entire mt genome size of *Ancylostoma* sp. from other known hookworm species is also due to longer rRNA sizes.

*Ancylostoma* sp. had an AT-rich region with a length of 261 bp and maximum A+T content of 90%. The placement of the AT-rich region of *Ancylostoma* sp. was between *trnA* and *trnP*, which is consistent with all hookworms [10, 48, 51]. Although the function of the AT-rich region has not yet been explored, it is believed to be the epicenter for the the initiation of gene replication and transcription [15]. The SNCR in *Ancylostoma* sp. was larger than that of other hookworms in the families Ancylostomatinae and Bunostominae [10, 49–51]. However, the position of the SNCR in the mt genome was identical to that of other *Ancylostoma* species [50, 53]. The LNCR in *Ancylostoma* sp. (106 bp) was larger than that in most hookworm species, with the exception of *A. tubaeforme* (107 bp) and *B. phlebotomum* (108 bp) [48, 50]. Previous studies showed that the NCR contained repeat sequences of TTTTA in *A. caninum* and *A. ceylanicum*; TATATTTAGT in *A. tubaeforme*; and TTTG in *A. doudenale* [48]. However, none of these repeat sequences were found in the NCR of *Ancylostoma* sp. Thus, the NCR of *Ancylostoma* sp. is an important region that differentiates this species from other *Ancylostoma* species.

Phylogenetic analyses of 18S rRNA and the complete mt genome showed that the *Ancylostoma* sp. clustered with *A. ceylanicum*, *A. caninum*, *A. tubaeforme* and *A. duodenale* in the subfamily Ancylostomatinae. However, some differences in the size of the mt genome, codon usage in PCGs, NCR sequences and tRNA secondary structures of the *Ancylostoma* sp. mt genome were helpful to differentiate it from other *Ancylostoma* species. Based on these results, we believe that this is a novel *Ancylostoma* species in the family Ancylostomatidae.

## Conclusions

We characterized the complete mt genome of an *Ancylostoma* sp. isolated from the Sunda pangolin (*Manis javanica*) by Illumina sequencing of total DNA. Amplified 18S rRNA and mt genome data identified this *Ancylostoma* sp. as a novel species in the Ancylostomatidae family. The identification of this novel mtDNA sequence enriches our knowledge of mt genomes in the Ancylostomatidae family.

## Supplementary Information

**Additional file 1: Figure S1.** Predicted secondary structure of tRNA genes in the *Ancylostoma* sp. mt genome, **Table S1.** Retrieved 18S rRNAs in species of Strongylida and Ascaridida from GenBank, **Table S2.** The mt genome from species in Strongylida, **Table S3.** Nucleotide composition (%) of PCGs, entire mt genome, and skew value of *Ancylostoma* sp.

## Abbreviations

*atp**6* and *atp**8*: Genes coding ATPase subunits 6 and 8, respectively
*cox**1-3*: Cytochrome* c* oxidase subunits I–III
*cob*: Cytochrome b
MtDNA: Mitochondrial DNA
*nad**1-6* and *nad**4L*: NADH dehydrogenase subunits 1–6 and 4L, respectively
NCR: Non-coding region
PCG: Protein-coding gene
rRNA: Ribosomal ribonucleic acid
*rrn**S *and *rrn**L*: Small and large ribosomal subunits, respectively
tRNA: Transfer RNA
