# Supplementary material for: Molecular detection of a novel Ancylostoma sp. by whole mtDNA sequence from pangolin Manis javanica
Source: Parasit Vectors. 2022 Mar 2;15:70. doi: 10.1186/s13071-022-05191-0 (PMC8889679; doi:10.1186/s13071-022-05191-0)
Supplement: Supplementary file 1 — Additional file 1: Figure S1. Predicted secondary structure of tRNA genes in the Ancylostoma sp. mt genome, Table S1. Retrieved 18S rRNAs in species of Strongylida and Ascaridida from GenBank, Table S2. The mt genome from species in Strongylida, Table S3. Nucleotide composition (%) of PCGs, entire mt genome, and skew value of Ancylostoma sp. [file 13071_2022_5191_MOESM1_ESM.docx]

**Supplementary Tables and Figures**


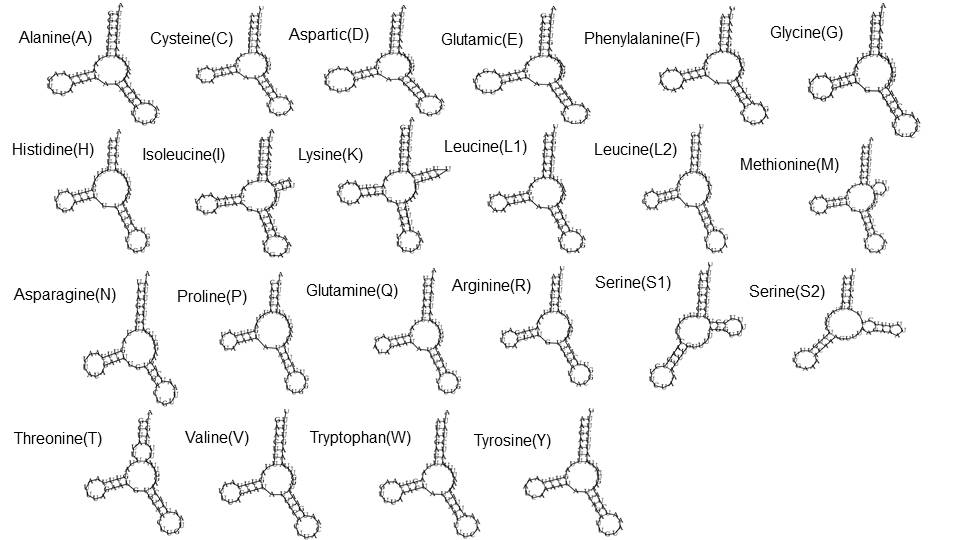


**Supplementary Figure S1.** Predicted secondary structure of tRNA genes in the *Ancylostoma* sp. mt genome.

**Supplementary Table S1.** Retrieved 18S rRNAs in species of Strongylida and Ascaridida from GenBank.

| **Family** | **Species** | **Size (bp)** | **Accession number** |
| --- | --- | --- | --- |
| Ancylostomatidae | *Necator americanus* | 1,737 | AJ920348.1 |
|  | *Ancylostoma caninum* | 1,737 | AJ920347.2 |
|  | *Ancylostoma duodenale* | 1,622 | EU344798.1 |
|  | ***Ancylostoma* sp.** | **1,681** | **MZ681936.1** |
| Trichostrongylidae | *Ostertagia ostertagi* | 1,738 | AJ920352.1 |
|  | *Ostertagia leptospicularis* | 1,738 | AJ920351.1 |
| Metastrongylidae | *Metastronylus elongatus* | 1,738 | AJ920363.2 |
|  | *Metastrongylus salmi* | 1,687 | AY295809.1 |
| Angiostrongylidae | *Angiostrongylus cantonensis* | 1,691 | AY295804.1 |
|  | *Angiostrongylus costaricensis*  *Angiostrongylus vasorum* | 1,759  1,738 | DQ116748.1  AJ920365.1 |
| Dictyocaulidae | *Dictyocaulus filaria* | 1,783 | AJ920362.1 |
|  | *Dictyocaulus viviparus* | 1,763 | AJ920361.1 |
| Toxocaridae | *Toxocara cati* | 1,798 | EF180059.1 |
|  | *Toxocara canis* | 1,754 | U94382.1 |

**Supplementary Table S2**. The mt genome from order of Strongylida

| **Family** | **Species** | **Size (bp)** | **Accession number** |
| --- | --- | --- | --- |
| Ancylostomatidae | *Ancylostoma duodenale* | 13,721 | AJ417718.1 |
|  | *Ancylostoma duodenale* | 13,710 | AP017676.1 |
|  | *Ancylostoma tubaeforme* | 13,730 | KY070315.1 |
|  | *Ancylostoma caninum* | 13,702 | AP017673.1 |
|  | *Ancylostoma caninum* | 13,717 | FJ483518.1 |
|  | *Ancylostoma ceylanicum* | 13,655 | AP017674.1 |
|  | *Ancylostoma ceylanicum* | 13,660 | KY640299.1 |
|  | *Necator americanus* | 13,606 | AJ556134.1 |
|  | *Bunostomum phlebotomum* | 13,790 | FJ483517.1 |
|  | *Bunostomum phlebotomum* | 13,803 | KF011546.1 |
|  | *Bunostomum trigonocephalum* | 13,764 | JQ234674.1 |
|  | *Bunostomum trigonocephalum* | 13,771 | KF255998.1 |
|  | ***Ancylostoma* sp.** | **13,757** | **MZ665481.1** |
| Chabertiidae | *Chabertia erschowi* | 13,705 | KF660603.1 |
|  | *Chabertia ovina* | 13,682 | GQ888721.1 |
|  | *Oesophagostomum dentatum* | 13,751 | AP017691.1 |
|  | *Oesophagostomum quadrispinulatum* | 13,681 | FM161883.1 |

**Supplementary Table S3.** Nucleotide composition (%) of protein coding genes (PCGs), entire mt genome, and skew value of *Ancylostoma* sp.

| **Gene** | **A** | **G** | **C** | **T** | **A+T (%)** | **G+C (%)** | **AT skew** | **GC skew** |
| --- | --- | --- | --- | --- | --- | --- | --- | --- |
| *cox*1 | 22 | 22 | 10 | 46 | 68 | 32 | -0.32 | 0.36 |
| *cox*2 | 26 | 20 | 8 | 46 | 72 | 28 | -0.24 | 0.42 |
| *nad*3 | 24 | 18 | 2 | 56 | 80 | 20 | -0.38 | 0.78 |
| *nad*5 | 26 | 18 | 6 | 50 | 76 | 24 | -0.29 | 0.48 |
| *nad*6 | 25 | 18 | 4 | 53 | 78 | 22 | -0.36 | 0.61 |
| *nad*4L | 25 | 16 | 3 | 56 | 81 | 19 | -0.35 | 0.65 |
| *nad*1 | 24 | 18 | 8 | 50 | 74 | 26 | -0.32 | 0.36 |
| *atp*6 | 27 | 17 | 6 | 50 | 77 | 23 | -0.29 | 0.44 |
| *nad*2 | 25 | 15 | 5 | 55 | 80 | 20 | -0.34 | 0.48 |
| *cob* | 25 | 17 | 8 | 50 | 75 | 25 | -0.31 | 0.33 |
| *cox*3 | 23 | 20 | 9 | 48 | 71 | 29 | -0.33 | 0.38 |
| *nad*4 | 25 | 15 | 7 | 53 | 78 | 22 | -0.35 | 0.35 |
| LNCR | 38 | 11 | 7 | 44 | 82 | 18 | -0.06 | 0.20 |
| SNCR | 36 | 19 | 3 | 42 | 78 | 22 | -0.04 | 0.67 |
| AT-Rich | 46 | 6 | 4 | 44 | 90 | 10 | 0.00 | 0.21 |
| Cmtg | 27 | 17 | 7 | 49 | 76 | 24 | -0.26 | 0.41 |
| EPCG | 25 | 18 | 7 | 50 | 75 | 25 | -0.32 | 0.42 |
| EtRNA | 36 | 14 | 6 | 44 | 80 | 20 | -0.08 | 0.36 |
| ErRNA | 35 | 15 | 6 | 44 | 79 | 21 | -0.11 | 0.40 |
| ENCR | 41 | 10 | 4 | 45 | 86 | 14 | -0.02 | 0.40 |

Cmtg: Complete mitochondrial genome, E: entire (PCG, tRNA, rRNA, NCR)
